# Associations of cachexia and frailty with amyotrophic lateral sclerosis

**DOI:** 10.1038/s41598-025-89080-3

**Published:** 2025-02-05

**Authors:** Tracy L. Peters, Weihong Qiu, Haomin Yang, Wuqing Huang, Yizhen Hu, Zhangyu Zou, Weimin Ye

**Affiliations:** 1 Department of Neurology, Union Hospital, Fujian Medical University, Fuzhou, 350001 Fujian China; 2 Department of Epidemiology and Health Statistics, School of Public Health, Fujian Medical University, Fuzhou, 350122 Fujian China; 3 Institute of Population Medicine, School of Public Health, Fujian Medical University, Fuzhou, 350122 Fujian China; 4 Department of Medical Epidemiology and Biostatistics, Karolinska Institutet, Stockholm, 171 77 Sweden; 5 Fujian Provincial Key Laboratory of Environmental Factors and Cancer, Fujian Medical University, Fuzhou, 350000 Fujian China

**Keywords:** Amyotrophic lateral sclerosis, Epidemiology, Frailty, Cachexia, Diseases, Cardiovascular diseases, Neurological disorders

## Abstract

In the present study, we investigated the associations of cachexia (loss of muscle, weight and fat) and frailty (loss of weight and muscle) status with the risk of developing amyotrophic lateral sclerosis, because these specific terms are rarely used in this research area. In this prospective study, we extracted cachexia and frailty status from the UK Biobank cohort to study the associations of these conditions (as determined via international classification of disease-10 codes) with amyotrophic lateral sclerosis. There was a greater risk of developing amyotrophic lateral sclerosis among individuals with cachexia and frailty status after adjusting for age, sex, income (pounds), body mass index, UK Biobank centers and smoking status. Among individuals with frailty status: a grip strength of < 21 kg, a slow walking speed, and exhaustion (more than half the days or nearly every day) increase the risk of developing amyotrophic lateral sclerosis. We believe that studying cachexia and frailty status can be used to help define and treat amyotrophic lateral sclerosis.

## Introduction

Amyotrophic lateral sclerosis (ALS) is characterized by degeneration of upper and lower motor neurons leading to progressive muscle weakness, muscle atrophy, fasciculation, hyperreflexia, muscle stiffness, and muscle cramping^1^. Patients with bulbar onset commonly present with dysarthria or dysphagia. Sporadic ALS does not show evidence of inheritance, although it shares several Familial ALS (fALS) genes (~ 5%)^2^. Familial ALS, accounting for 10% of ALS cases, is characterized by the presence of approximately 40 genes that impact the etiology and pathogenesis of ALS^2^. Scientists have reported several main mutations in fALS, including C9ORF72 (Chromosome 9 Open Reading Frame 72) (40%), SOD1 (Cu-Zn superoxide dismutase 1) (12%), FUS (fused in sarcoma) (4%) and TARDBP (transactive response DNA binding protein 43 kDa) (4%)^2^. ALS involves mainly the motor system and, to a lesser extent, the extra-pyramidal system^1^. Several non-motor symptoms have been reported in ALS, among which fatigue was the most common (81.8%), other symptoms include: sleep disorders (48.5%), sexual problems (24.2%), sialorrhea (48.5%), defecation disorders (57.6%), and pain (60.6%)^3^. Approximately 40% of ALS patients have mild to moderate neuropsychological damage^4^. Cognitive impairment occurs in individuals with ALS with loss of executive dysfunction (attention, memory, inhibition, and language deficits)^4^. Frontotemporal dementia (FTD) (present in approximately 15% of ALS patients) affects the frontal and anterior temporal lobes, leading to language impairments, executive function impairments and behavioral changes^1^.

Cachexia is characterized by severe weight loss with low muscle mass; fat mass can also be lost, and these changes cannot be altered with just nutritional intake interventions^5^. Cachexia is common in cancer, and there is an ongoing loss of skeletal muscle mass that affects function^6^. The disease can expand to affect other tissues (heart and bone) and can lead to symptoms of fatigue, anorexia, and anhedonia^6^.

Frailty is defined as the presence of three or more of the following symptoms: low grip strength, slow gait speed, low physical activity levels, exhaustion, and unexpected weight loss^7^. One paper examined the association between frailty status and the risk of developing ALS^8^, but no association between biological aging and the risk of developing ALS was detected. Frailty usually occurs in older individuals, and the prevalence of frailty is expected to increase^9^. Frailty status is associated with an increased risk of falls, hospitalizations, and mortality^9^. Older adults are at greater risk of developing frailty if they have comorbidities, bad eating behaviors, or low socioeconomic status or if they are sedentary^9^.

Cachexia and frailty status are typically assessed in patients with cardiovascular disease (CVD) or cancer^6,10^ and are rarely studied in individuals with ALS^8,10–13^. In terms of CVD, several studies have been conducted to assess the effects of this condition on individuals with ALS^14–17^, and they have involved the investigation of associations^14^, prescriptions of cardiovascular medications^15^, hospital-based populations^16^, and self reported clinical characteristics^17,18^.

The terms cachexia and frailty are rarely used in the ALS literature, but information on these conditions among patients with ALS could be useful for identifying treatment options and modifiable lifestyle factors. Therefore, we obtained cachexia and frailty status data from the UK Biobank to study the associations of these conditions with ALS identified by the International Classification of Diseases (ICD)-10 codes.

## Results

**Table 1:** Basic characteristics of the UK Biobank participants.

	General Data No.^b^ (%)	ALS Cases No.^b^ (%)
Total Population	501,697 (99)	630 (01)
Age, continuous, median (range)	58 years old (36)	63 years old (30)
BMI^a^, continuous, median (range)	27 (63)	27 (33)
Sex		
Male	228,689 (46)	352 (56)
Female	273,008 (54)	278 (44)
Income Level		
Less than 18,000£	97,017 (19)	145 (23)
18,000 to 30,999£	107,981 (22)	151 (24)
31,000 to 51,999£	110,617 (22)	125 (20)
52,000 to 100,000£	86,169 (17)	71 (11)
Greater than 100,000£	22,901 (05)	22 (03)
Cannot Answer	71,019 (14)	100 (16)
Missing	5993 (01)	16 (03)
Smoking Status		
Prefer Not To Answer	2054 (0)	2 (0)
Never	273,118 (54)	312 (50)
Previous	172,742 (34)	250 (40)
Current	52,893 (11)	65 (10)

^a^Body Mass Index (BMI)

^b^Number

During the follow-up of 6,666,183 person-years, 630 incident ALS cases were identified in the cohort, corresponding to an incidence rate of 9.45/100,000 person-years. The descriptive information for the UK Biobank data are provided in Table 1. Patients with ALS were most likely to be within these three income (£) brackets: less than 18,000£ (145 (23%) ALS patients), 18,000 to 30,999£ (151 (24%) ALS patients), and 31,000 to 51,999£ (125 (20%) ALS patients) (Table 1). A low percentage of ALS patients were current smokers (10%, *n* = 65) (Table 1).

**Fig. 1:**
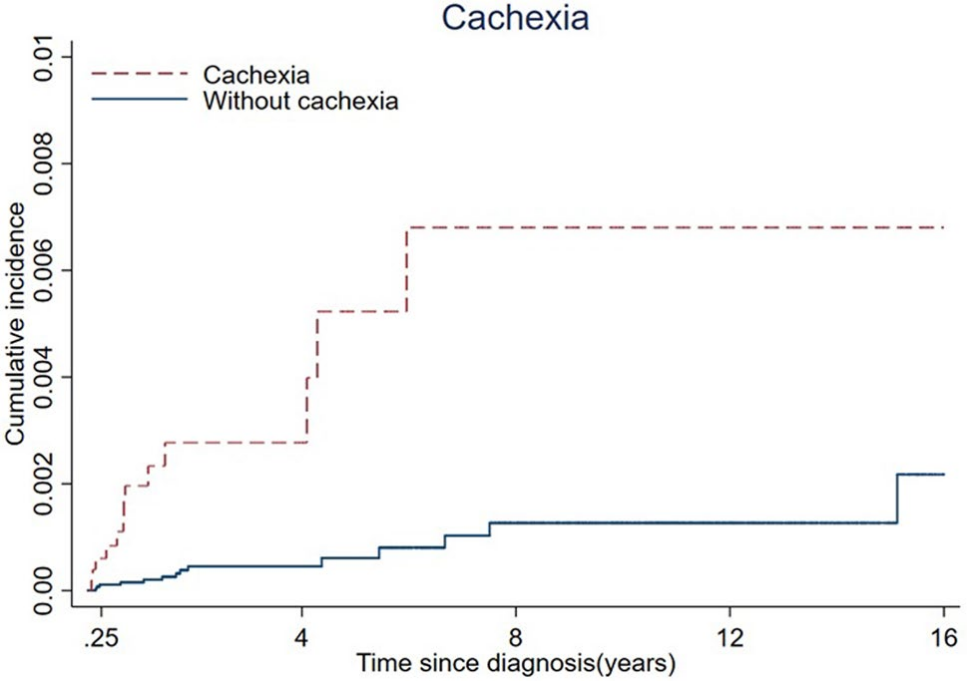
The Cumulative Incidence of ALS by Cachexia Status.

**Fig. 2:**
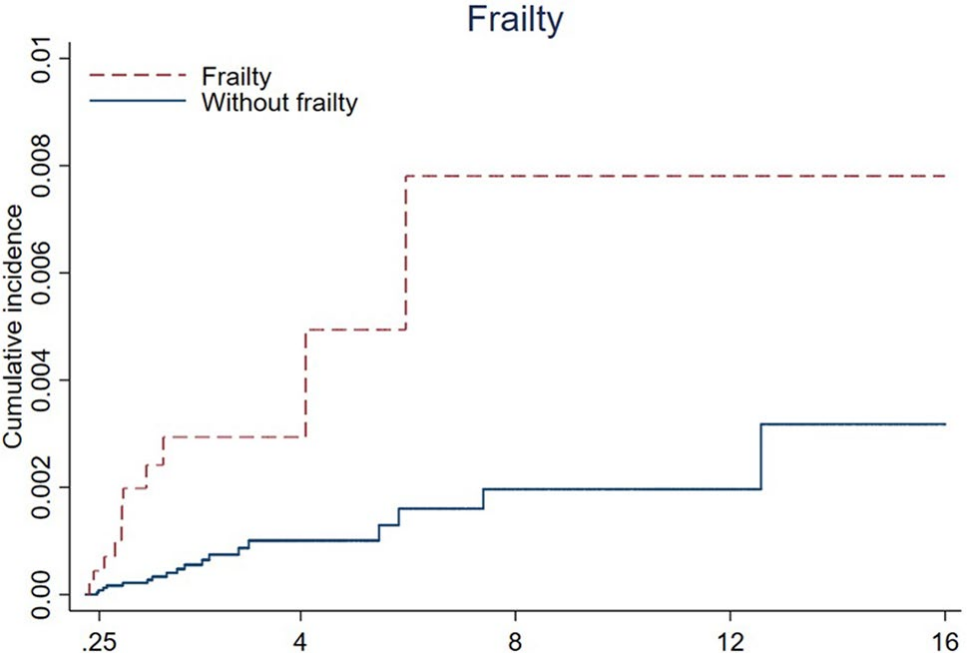
The Cumulative Incidence of ALS by Frailty Status.

The cumulative incidence rates of ALS caused by cachexia and frailty are plotted in Figs. 1 and 2.

As shown in Table 2, the risk of developing ALS was significantly positively associated with cachexia status (HR: 4.85, 95% CI: 2.50–9.41) and frailty status (HR: 6.42, 95% CI: 3.29–12.53) after adjusting for age, sex, income (£), BMI, UKB center and smoking status. We also found that patients with ALS were more likely to have grip strength < 21 kg (HR: 1.28, 95% CI: 1.02–1.61), a slow walking pace (HR: 1.37, 95% CI: 1.02–1.83), and exhaustion for more than half the days or nearly every day (HR: 4.89, 95% CI: 3.08–7.75), after adjusting for age, sex, income (£), BMI, UKB center and smoking status.

**Table 2:** Associations of Cachexia and Frailty Status with the Risk of Developing ALS

	HR^i^ (95% CI^j^)^a^	HR^i^ (95% CI^j^)^b^ Sex	HR^i^ (95% CI^j^)^c^ Income (£)	HR^i^ (95% CI^j^)^d^ BMI ^k^	HR^i^ (95% CI^j^)^e^ Smoking	HR^i^ (95% CI^j^)^f^ All Adjustments
Cachexia						
No	Reference	Reference	Reference	Reference	Reference	Reference
Yes	5.17 (2.76–9.71)	5.04 (2.69–9.47)	5.01 (2.58–9.73)	4.83 (2.49–9.38)	5.11 (2.73–9.61)	4.85 (2.50–9.41)
Frailty						
No	Reference	Reference	Reference	Reference	Reference	Reference
Yes	6.30 (3.24–12.27)	6.26 (3.22–12.18)	6.11 (3.02–12.29)	6.57 (3.37–12.80)	6.21 (3.19–12.09)	6.42 (3.29–12.53)
Frailty Status						
Other ^g^	Reference	Reference	Reference	Reference	Reference	Reference
Decreased Activity and Body Mass^h^	1.01 (0.86–1.2)	1.2 (0.99–1.4)	1.03 (0.86–1.22)	1.01 (0.85–1.19)	1.02 (0.86–1.20)	1.16 (0.97–1.39)
Grip Strength (kg)						
≥ 22	Reference	Reference	Reference	Reference	Reference	Reference
< 21	0.96 (0.81–1.15)	1.41 (1.12–1.76)	0.96 (0.79–1.15)	0.93 (0.77–1.11)	0.97 (0.81–1.16)	1.28 (1.02–1.61)
Walking Speed						
Other	Reference	Reference	Reference	Reference	Reference	Reference
Slow Pace	1.29 (0.99–1.70)	1.31 (0.99–1.72)	1.34 (1.02–1.76)	1.31 (0.98–1.75)	1.28 (0.98–1.68)	1.37 (1.02–1.83)
Weight Loss						
Other	Reference	Reference	Reference	Reference	Reference	Reference
Weight Loss	0.99 (0.78–1.25)	0.99 (0.79–1.26)	0.96 (0.76–1.23)	0.95 (0.75–1.21)	0.98 (0.78–1.25)	0.94 (0.73–1.19)
Exhaustion						
Other	Reference	Reference	Reference	Reference	Reference	Reference
More than half the days or nearly every day	4.17 (2.66–6.53)	4.36 (2.78–6.84)	4.28 (2.72–6.73)	4.49 (2.86–7.08)	4.22 (2.69–6.62)	4.89 (3.08–7.75)
Physical Activity Status						
Active	Reference	Reference	Reference	Reference	Reference	Reference
No-Low Activity	1.08 (0.90–1.29)	1.11 (0.92–1.33)	1.09 (0.91–1.30)	1.08 (0.90–1.29)	1.08 (0.89–1.29)	1.12 (0.93–1.35)

^a^Adjusted for age

^b^Adjusted for age and sex

^c^Adjusted for age and income (£)

^d^Adjusted for age and BMI ^k^

^e^Adjusted for age and smoking

^f^Adjusted for age, sex, income (£), BMI ^k^, UK Biobank centers and smoking status

^g^Other walking speed, other weight loss, other exhaustion, grip strength ≥ 22 kg, and active status

^h^Slow walking pace, weight loss, more than half the days or nearly every day, grip strength < 21 kg, and no-low activity status

^i^Hazard Ratio

^j^Confidence Interval

^k^Body Mass Index

^i^Kilogram

**Table 3:** Associations of Cachexia and Frailty Status with the Risk of Developing ALS

	General Data	ALS Cases	≤Age 65^a^	> Age 65^a^	Male ^a^	Female ^a^
	No.^d^ (%)	No.^d^ (%)	HR^e^ (95% CI^f^)	HR^e^ (95% CI^f^)	HR^e^ (95% CI^f^)	HR^e^ (95% CI^f^)
Cachexia						
No	496,300 (99)	620 (98)	Reference	Reference	Reference	Reference
Yes	5397 (01)	10 (02)	2.41 (0.60–9.69)	6.41 (2.82–14.61)	3.48 (1.29–9.38)	6.27 (2.31–17.01)
Frailty						
No	496,877 (99)	621 (98)	Reference	Reference	Reference	Reference
Yes	4820 (01)	9 (02)	5.38 (1.71–16.95)	7.12 (3.12–16.29)	4.61 (1.70–12.50)	9.25 (3.75–22.79)
Frailty Status						
Other ^b^	192,468 (38)	232 (37)	Reference	Reference	Reference	Reference
Decreased Activity and Body Mass^c^	308,893 (62)	398 (63)	1.18 (0.94–1.48)	1.15 (0.84–1.56)	1.11 (0.89–1.39)	1.28 (0.93–1.75)
Grip Strength (kg)^g^						
≥ 22	361,337 (72)	434 (69)	Reference	Reference	Reference	Reference
< 21	138,003 (28)	194 (31)	1.24 (0.93–1.64)	1.40 (0.94–2.09)	1.79 (1.18–2.71)	1.13 (0.87–1.47)
Walking Speed						
Other	458,092 (91)	556 (88)	Reference	Reference	Reference	Reference
Slow Pace	40,814 (08)	66 (10)	1.65 (1.15–2.37)	0.99 (0.60–1.65)	1.68 (1.16–2.43)	0.99 (0.62–1.61)
Weight Loss						
Other	425,072 (85)	536 (85)	Reference	Reference	Reference	Reference
Weight Loss	75,706 (15)	93 (15)	0.95 (0.70–1.28)	0.91 (0.59–1.40)	0.81 (0.57–1.15)	1.09 (0.78–1.55)
Exhaustion						
Other	147,945 (29)	66 (10)	Reference	Reference	Reference	Reference
More than half the days or nearly every day	19,137 (04)	30 (05)	5.29 (3.19–8.75)	3.45 (1.06–11.16)	5.62 (3.01–10.50)	4.27 (2.16–8.45)
Physical Activity Status						
Active	300,954 (60)	362 (58)	Reference	Reference	Reference	Reference
No-Low Activity	160,831 (32)	210 (33)	1.08 (0.86–1.35)	1.21 (0.88–1.66)	1.23 (0.96–1.57)	0.99 (0.75–1.31)

^a^Adjusted for age, sex, income (£), Body Mass Index, UK Biobank Center, and smoking status

^b^Other walking speed, other weight loss, other exhaustion, grip strength ≥ 22 kg, and active status

^c^Slow walking pace, weight loss, more than half the days or nearly every day, grip strength < 21 kg, and no-low activity status

^d^Number

^e^Hazard Ratio

^f^Confidence Interval

^g^Kilogram

With respect to frailty status, we found that among males, a grip strength < 21 kg (HR: 1.79, 95% CI: 1.18–2.71), a slow walking pace (HR: 1.68, 95% CI: 1.16–2.43), and exhaustion more than half the days or nearly every day (HR: 5.62, 95% CI: 3.01–10.50) were positively associated with the risk of developing ALS (Table 3). Additionally, we found that a slow walking pace (HR: 1.65, 95% CI: 1.15–2.37) and exhaustion for more than half the days or nearly every day (HR: 5.29, 95% CI: 3.19–8.75) were positively associated with the risk of developing ALS in individuals aged ≤ 65 years (Table 3).

The sensitivity analyses with 1-year and 3-year exposure lags still revealed statistically significant associations of cachexia and frailty status with the risk of developing ALS. Three-year time lag exposure to cachexia (565 ALS patients without cachexia, 3 ALS patients with cachexia) and frailty (566 ALS patients without frailty, 2 ALS patients with frailty) was associated with the risk of developing ALS (cachexia, HR: 3.89 (95% CI: 1.24–12.12) and frailty, HR: 4.78 (1.19–19.22)), whereas 5-year time lag exposure to cachexia and frailty was not associated with the risk of developing ALS (cachexia, HR: 1.77 (95% CI: 0.25–12.59) and (frailty, HR: 3.65 (0.51–26.01)).

## Discussion

In this study, we found that cachexia and frailty status were associated with a greater risk of developing ALS, and we used ICD codes for patients and compared them with those of normal controls. This information provides an opportunity to expand the understanding of the disease and its definition. Most cachexia and frailty studies are basic science studies^8,10,11^ or review papers^13,19^. We performed stratified analyses for frailty status and found that grip strength < 21 kg, a slow walking pace, and exhaustion for more than half the days or nearly every day are associated with an increased risk of developing ALS.

There are a limited number of studies that have examined the association between frailty status and the risk of developing ALS, including two studies of genes^7,11^ and a study about the end of life^12^. Briefly, SOD1-ALS mutations have been reported to be associated with frontal lobe frailty^11^. The frontal lobe is located in the brain and is associated with cognitive control^20^. Frontal lobe frailty or frontal lobe damage causes executive function deficits and control problems^20^. With increased attention to frailty as a disease, there are various frailty subtypes, including social frailty, nutritional frailty and cognitive frailty^9^. A different ALS study investigated the cognitive aspects of gait abnormalities and falls with a wearable gait analysis device^21^. They reported that mild cognitive impairment was associated with exaggerated gait and a greater number of falls at 3 months in patients with ALS^21^. Like their paper, our paper revealed that ALS patients with frailty (slow walking pace) had an increased risk of developing ALS^21^. Our study investigated frailty status, which is significantly associated with the risk of developing ALS in general and in males. Studies of frailty have shown a greater prevalence of frailty in women, and the prevalence increases with age^9^. Surprisingly, in individuals with ALS, the risk of developing ALS increases by the age of 80 years in men (1 in 300)^22^.

Another study examined symptoms of ALS (demographic and clinical factors, frailty, and acute healthcare utilization) at the end of life^12^. Studies have shown that in patients with ALS, frailty status is associated with greater numbers of emergency room visits and hospital deaths^12^. Frailty can present as signs, symptoms, disabilities, and abnormal test results^9^, and it is associated with healthcare utilization in individuals with ALS; however, specialized palliative care is equal in terms of sex, frailty status, socioeconomic status and age (< 75 years or not)^12^. Progressive muscle atrophy (weight loss) and poor oral intake (poor nutritional status) are associated with rapid ALS disease progression^19^. Nutritional assessment and weight management intervention occurs fairly quickly in the ALS referral process^19^.

It is important to examine the breakdown of symptoms occurring in individuals with frailty. Frailty classifications exist;^23^ however, a widely accepted frailty phenotype includes the presence of three out of five symptoms, including weight loss, exhaustion, physical activity, walking speed and grip strength^7^. One paper on the association between frailty status and the risk of developing ALS reported no associations between the two conditions, and frailty status was considered an indicator of biological aging^8^. However, in that study, frailty status was calculated on the basis of the total number of deficits and the number of patient deficits divided by 49 possible deficits^8^. In men and in general, we found an increased risk of developing ALS among those who had grip strength < 21 kg, a slow walking pace, and exhaustion on more than half the days or nearly every day. Frailty status has proven to be a good method for identifying elderly people at risk for symptoms, diseases and death^24^. There are some discrepancies in the results of frailty research, as molecular or ideal biomarkers in frailty research have not been properly identified; this gap warrants further exploration^24^. Mendelian randomization analyses have been performed to assess the associations of Alzheimer’s disease, Parkinson’s disease, ALS, and neurological tumors with biological aging indicators (frailty status, telomere length, facial aging, and epigenetic aging clock acceleration)^8^, but no evidence of a relationship between the risk of developing ALS and biological aging^8^.

The genetics of ALS are known to involve specific clinical phenotypes^2^. C9ORF72 is associated with changes in cognition and behavior^2^. SOD1 is associated with motor degeneration, which may have an impact on cachexia and frailty status because of the associated loss of muscle^2^. Dementia presents in ALS patients with FUS and TARDBP^2^. A younger ALS onset can be found in patients with FUS^2^. We found that a slow walking pace was associated with the risk of developing ALS in patients aged ≤ 65 years.

Cachexia can be described by a poor clinical outcome, loss of muscle and loss of fat mass^8^. The literature on the association between cachexia and the risk of developing ALS is limited and unclear. Cachexia and ALS can be found together in review articles, with a discussion of the word in relation to malnutrition^13,19^. A further problem is that, clinically, cachexia can resemble malnutrition or frailty^6^. Cachexia may occur in the early course of ALS without the presence of bulbar symptoms. Cachexia in individuals with ALS has been associated with oxidative stress and malnutrition and has been identified as a complex metabolic syndrome^13^. Malnutrition accounts for approximately 50% of ALS cases^19^. In our study, we found that cachexia was associated with an increased risk of developing ALS. Moreover, we found that this association was unaffected by age (≤ 65 years and > 65 years). As seen in the literature, cachexia usually occurs at the end stage of ALS, and changes in diet (i.e., achieving nutritionally adequacy) to include antioxidant and anti-inflammatory components are recommended^19^.

There are several basic science papers that discuss the association between cancer cachexia and the risk of developing ALS^10,25^. Muscle wasting has similar mechanisms in cancer cachexia and ALS at the catabolic level^10^. p97 muscle proteins are degraded during atrophy by denervation^10^. Cecconi et al.^10^ reported that the p97-Nploc4 complex might serve as a novel drug target, as it contributes to muscle atrophy in individuals with cancer cachexia and ALS. Atrophy of the skeletal muscle of the diaphragm and/or heart can cause death in both diseases^10^. In another study, Pasetto et al.^25^ developed a noninvasive procedure, microcomputed tomography (micro-CT), to investigate hind limb muscle wasting in transgenic SOD1^G93A^ and colon adenocarcinoma C26-bearing mice. The authors found that they could detect reduced muscle content by micro-CT in both diseases.

ALS occurs in a population of people with greater cardiovascular fitness^18^. For patients with ALS, ‘physical fitness’ is observed repeatedly by clinicians^18^. In terms of frailty status in our study, these indicators, namely, poor grip strength (< 21 kg), slow walking pace and daily exhaustion, were associated with an increased risk of developing ALS in men. Coronary heart disease (CHD) could be an indicator of reduced cardiovascular fitness^18^. Cachexia and frailty are terms that are used in the heart disease literature^5^. Studies have been conducted on the association between CVD and the risk of developing ALS^16–18^. A Danish nested case‒control study examining associations of ALS diagnosis with CVD status and CVD-specific hospital admissions (ischemic heart disease and atherosclerosis) was performed. Three years prior to the index date, the team detected a significantly greater risk of developing ALS among patients with atherosclerosis^14^. A population-based case‒control study was performed in the United States and investigated the associations of CVD medication prescriptions with the risk of developing ALS^15^. Inverse associations with ALS risk were found among those exposed to CVD medications, including ACE inhibitors, beta blockers, and calcium channel blockers^15^.

The autonomic nervous system (ANS) controls the heart, and dysfunction arises from disorders of the autonomic nerves or cardiac disease^26^. ANS control of the heart is controlled by transmitted afferent neural impulses, extracardiac intrathoracic ganglia, to the spinal cord and to the brainstem^26^. The ANS has a presentation similar to that of cachexia, and its process may affect the loss of muscle, weight and fat. A few studies related to ALS have been conducted on autonomic dysfunction^27–29^. A study reported two key findings, one being that ALS autonomic dysfunction is frequent but modest and the second finding is when upper motor neurons are affected autonomic deficits are greater^29^. One study investigated the ANS in ALS patients by evaluating the sympathetic skin response (SSR). The SSR is a test that records sympathetic autonomic function, and the test records the change in electrical signaling of active sweat glands on the skin^27^. Researchers have reported that the risk of developing ALS is associated with an abnormal SSR, reaffirming that the ANS is involved in ALS development^27^. Another study investigated the relationship between autonomic dysfunction and disease survival in patients with ALS^28^. These authors reported that autonomic symptoms are associated with the risk of developing ALS and that symptoms progress over time, especially in bulbar-onset patients^28^. Cutaneous innervation, which refers to the sensory nerve fibers to the skin, could be a mechanism that could be affected when cachexia and/or frailty occur^30^. An ALS study investigated cutaneous innervation and clinical features^31^. The authors reported that peripheral sensory involvement occurs alongside motor disability^31^.

The endocannabinoid system (ECS) and metabolism, like the ANS, may be involved in cachexia, frailty, and ALS. The ECS is a lipid system that modulates appetite control, energy balance, endocrine homeostasis, and metabolism^32^. Carbohydrates, fat, and proteins are controlled by the liver and process metabolism^33^. Metabolism maintains energy homeostasis that encompasses chemical reactions and pathways^33^. Recent research has shown that among individuals with obesity, the ECS regulates energy metabolism, food intake and inflammatory responses^32^. Endocannabinoid (eCB) signaling is altered in patients with ALS and may have an impact on the disease^32^. Few studies on this topic have been conducted in humans, and few studies have been conducted in ALS patients to determine the associations between the serum levels of eCB and disease status^34^. A previous study revealed changes in the levels of eCB in individuals with ALS^34^.

The use of these two definitions (cachexia and frailty) could expand treatment options and clinical perspectives. Only two drugs have been approved for the treatment of ALS: riluzole and edaravone^35^. In one study, researchers evaluated the risk of developing ALS associated with the use of certain prescription drugs for other indications from U.S. Medicare^35^. They identified 10 drugs that could lower the risk of developing ALS^35^. These 10 drugs included drugs used for CVD, hypertension, and diabetes^35^. Cardiac cachexia is usually treated by nutrition and exercise; however, medically, it can be treated by neurohormonal blockade, appetite stimulants, anabolic hormones, immunomodulatory agents, physical exercise regimens and reducing intestinal bacterial translocation^36^. Cardiac cachexia treatment largely focuses on preserving muscle mass^36^.

Our study revealed that cachexia and frailty could increase a person’s risk of developing ALS. We believe this provides information for defining and potentially treating ALS. The frailty status overall defined in our study did not provide any new information; one would have to have at least three of the five criteria. The clinical diagnoses (which vary in accuracy and among healthcare providers) and classification of ALS often come from the El Escorial Criteria and revisions and ICD codes^37^. We use ICD codes to define frailty and cachexia. The ICD is a universal health statistics coding system^37^. The use of ICD codes can be limiting, and the diagnoses and diagnostic tools can introduce misclassification and underreporting, which introduces bias. A detailed clinical or genetic study could be conducted to be more thorough. The diagnosis of ALS is usually determined by clinical findings, electromyography results, and the exclusion of other diseases or mimics^22^. The use of frailty status provides insights into the breakdown of frailty and its possible causes associated with the risk of developing ALS.

## Methods

### Study population

The UK Biobank is a prospective cohort study involving approximately 500,000 participants conducted from 2006 to 2010 in the UK. This study included individuals aged 37 to 73 years who were recruited from 22 active assessment centers^38,39^. There were 228,689 (46%) participants and 352 (56%) ALS patients who were males and 273,008 (54%) participants and 278 (44%) ALS patients who were females in the UK Biobank. A baseline assessment consisted of questionnaires and verbal interviews that included information on lifestyle, health-related information, and sociodemographic data^38^. Biological samples were collected along with physical measurements.

All the participants provided informed consent to participate in the study. The UK Biobank was approved by the National Health Service (NHS), North West Multicenter Research Ethics Committee (Ref: 11/NW/0382, 17 June 2011), and the National Information Governance Board for Health and Social Care^38,39^. All the experimental methods were performed in accordance with relevant guidelines and regulations.

## Follow-Up

The follow-up started from the date of attendance at the UK biobank cohort and ended on the 31st of October, 2022, for England; 31st of August, 2022, for Scotland; and 31st of May, 2022, for Wales, considering the coverage of NHS registers for different regions. The follow-up ended at the date of death, loss to follow-up, date of ALS diagnosis, or end of follow-up.

## Diagnosis of cachexia and frailty

The inpatient hospitalization data from the ICD-10 were linked to the UK Biobank. The diagnosis of cachexia was determined by using the following ICD-10 codes: R64, E86, E23, A15, E88, F48, R54, N28, D73, and E03. The diagnosis of frailty was determined by using the following ICD-10 codes: R54, R41, and R53.

### Frailty status

We used the definition of frailty status created for the UK Biobank, which included the following criteria: weight loss, exhaustion, physical activity, walking speed, and grip strength (kg)^7^. An example of this procedure consisted of weight loss, which was self-reported as follows: “Compared with one year ago, has your weight changed?” (response: yes, lost weight = 1, other = 0)^7^.

Weight loss was defined as weight change compared with that one year prior (field ID 2306)^40^. Exhaustion was defined as feeling tired or having little energy over the last two weeks (field id 2080)^40^. Walking speed was defined as the usual walking pace (field ID 924)^40^. The grip strength (kg) measurement was defined as the lowest 20% at baseline^7,41^. Grip strength was defined as hand grip strength (kg) (left) and hand grip strength (kg) (right) (field IDs 46 and 47)^41^. In the UK Biobank, physical activity was defined as the frequency of heavy DIY in the last 4 weeks, the frequency of light DIY in the last 4 weeks, the frequency of walking for pleasure in the last 4 weeks, the frequency of other exercises in the last 4 weeks, and the frequency of strenuous sports in the last 4 weeks (field IDs 3637, 1011, 2624, 971, and 991)^40^.

We created a comprehensive frailty status indicator based on five different criteria^7^. We combined the reference categories of all the criteria, namely, other walking speed, other weight loss, other exhaustion, grip strength ≥ 22 kg, and active status to create a comprehensive reference group. The indicators of frailty status that could be associated with an increased risk of developing ALS consisted of slow walking pace, weight loss, exhaustion more than half the days or nearly every day, grip strength < 21 kg, and no-low activity status^7^.

## Diagnosis of ALS

ALS cases were obtained from the National Health Service Central Register and National Health Service Information Center. The inpatient hospitalization data containing ICD-10 codes were linked to the UK Biobank. The diagnosis of ALS was determined by using the following ICD-10 code: G12.

### Statistical analysis

Sex was categorized as male or female; income (£) level (annual household income (£) before tax) was categorized as less than 18,000£, 18,000 to 30,999£, 31,000 to 51,999£, 52,000 to 100,000£, greater than 100,000£, cannot answer and missing; body mass index (BMI) and age were analyzed as continuous; and smoking was categorized as preferring not to answer; never; previous; current. We used missing categories for missing income (£) and smoking data. The current literature analyzes BMI as a continuous variable^42–44^.

Cox proportional hazards models were used to calculate hazard ratios (HRs) and 95% confidence intervals (CIs), with attained age as a time scale. We controlled for age, sex, income (£), BMI, UKB center, and smoking status. We examined the associations between frailty and cachexia and the risk of developing ALS by considering the diagnoses of frailty and cachexia as time-dependent exposures, in which the exposed person-years were counted three months after the date of diagnosis to reduce the potential influence of reverse causality. In addition, frailty status was defined on the basis of examinations at baseline, and we performed further analyses according to the five criteria separately. Stratified analyses were performed by sex and age (≤ 65 years and > 65 years). We used age 65 as a cutoff, as this age can be associated with retirement age. There has been some concern that individuals have multiple conditions, and previous frailty studies have used age 65 as a cutoff to address this concern^6^. The cumulative incidence of ALS was plotted using the K‒M method for cachexia and frailty diagnosis during the follow-up time. For this analysis, 5 randomly selected participants were matched to patients with cachexia and frailty by age, sex and UKB center.

For the diagnosis of cachexia and frailty, we conducted a sensitivity analysis with a lag in the follow-up time from the date of diagnosis of one, three, or five years.

## Data Availability

The data that support the findings of this study are available from UK Biobank application #61083 but restrictions apply to the availability of these data, which were used under license for the current study, and so are not publicly available. Data are however available from the authors upon reasonable request and with permission of (Haomin Yang, haomin.yang@fjmu.edu.cn).
